# Intentional injury and violence in Cape Town, South Africa: an epidemiological analysis of trauma admissions data

**DOI:** 10.3402/gha.v8.27016

**Published:** 2015-06-12

**Authors:** Nadine Schuurman, Jonathan Cinnamon, Blake Byron Walker, Vanessa Fawcett, Andrew Nicol, Syed Morad Hameed, Richard Matzopoulos

**Affiliations:** 1 Department of Geography, Simon Fraser University, Burnaby, BC, Canada; 2 Department of Geography, University of Exeter, Exeter, UK; 3 Department of Surgery, University of Virginia, Charlottesville, VA, USA; 4 Trauma Unit, Groote Schuur Hospital, University of Cape Town, Cape Town, South Africa; 5 Faculty of Medicine, University of British Columbia, Vancouver, BC, Canada; 6 School of Public Health and Family Medicine, University of Cape Town, Cape Town, South Africa

**Keywords:** injury, violence, intentional, surveillance, trauma, Cape Town, South Africa

## Abstract

**Background:**

Injury is a truly global health issue that has enormous societal and economic consequences in all countries. Interpersonal violence is now widely recognized as important global public health issues that can be addressed through evidence-based interventions. In South Africa, as in many low- and middle-income countries (LMIC), a lack of ongoing, systematic injury surveillance has limited the ability to characterize the burden of violence-related injury and to develop prevention programmes.

**Objective:**

To describe the profile of trauma presenting to the trauma centre of Groote Schuur Hospital in Cape Town, South Africa – relating to interpersonal violence, using data collected from a newly implemented surveillance system. Particular emphasis was placed on temporal aspects of injury epidemiology, as well as age and sex differentiation.

**Design:**

Data were collected prospectively using a standardized trauma admissions form for all patients presenting to the trauma centre. An epidemiological analysis was conducted on 16 months of data collected from June 2010 to October 2011.

**Results:**

A total of 8445 patients were included in the analysis, in which the majority were violence-related. Specifically, 35% of records included violent trauma and, of those, 75% of victims were male. There was a clear temporal pattern: a greater proportion of intentional injuries occur during the night, while unintentional injury peaks late in the afternoon. In total, two-third of all intentional trauma is inflicted on the weekends, as is 60% of unintentional trauma. Where alcohol was recorded in the record, 72% of cases involved intentional injury. Sex was again a key factor as over 80% of all records involving alcohol or substance abuse were associated with males. The findings highlighted the association between violence, young males, substance use, and weekends.

**Conclusions:**

This study provides the basis for evidence-based interventions to reduce the burden of intentional injury. Furthermore, it demonstrates the value of locally appropriate, ongoing, systematic public health surveillance in LMIC.

Injury is a truly global health issue with massive societal and economic consequences. The recent edition of the Global Burden of Disease Study estimated that injury was responsible for more than 5 million annual global deaths (9.6% of total mortality) in 2010, a notable increase from the 1990 baseline of 4.1 million (8.8% of total mortality) (1). Mortality is, however, just the tip of the iceberg when it comes to injury; injury morbidity affects a much larger number of people, including those living with the consequences of injury, as well as their family members, friends, and community. In economic costs, those who are most economically productive in society are also more likely to get injured, meaning families of injured persons are often impoverished through lost income and the costs of health care (2). Countries are also acutely affected. Data on the direct costs to national health systems for patient care and indirect costs of injury (reduced productivity) at a national scale are estimates only and are typically not available in lower income settings; however, as an illustration of the economic burden of this health issue, the total direct and indirect cost of injury to Canada in 2004 was estimated at CAD 19.8 billion (3), and USD 403.7 billion to the United States in 2005 (4).

Injury prevention efforts have been shown to reduce the societal and economic costs of injury. The backbone of evidence-based injury prevention is *injury surveillance*, generally referred to as the systematic collection, analysis, interpretation, and dissemination of data on injury and its determinants. The analysis of current and appropriate data on injury has fuelled the development of effective prevention programmes and policies over the past several decades; however, these interventions have largely been confined to settings that have surveillance systems in place, mostly in high-income countries (HIC) (5). Limited surveillance in low- and middle-income countries (LMIC) has hindered the development of evidence-based efforts to combat injury in these settings (6), which also characteristically possess higher rates of injury than HIC. Europe provides a good example of the large contrast in injury rates according to development status; rates of injury are 45.6 per 100,000 overall for the continent's HIC, while in its LMIC, rates of injury are almost three times higher at 126.8 per 100,000 (5). A recent overview of injury in a global context (7) illustrated the huge injury burden in LMIC, and put forth an agenda for action to combat the problem in these countries. Of particular priority is the need to implement or enhance injury surveillance activities, and based on analysis of surveillance data, generate knowledge pertaining to injury epidemiology.

A constellation of conditions has intensified the burden of injury in Cape Town, the site of this study. Cape Town is one of the South Africa's six major metropolitan areas with a population of between 3.5 and 4.0 million people, almost two-thirds of the Western Cape provincial population (8). The city has been affected by large-scale internal migration from rural to urban areas in the last three decades and, more recently, cross-border migration due to civil unrest and economic instability in countries north of South Africa. According to 2011 census data, approximately 8% of Western Cape residents had moved from another province since 2001 (9). This has placed a considerable burden on already-stressed social infrastructure and services, particularly in communities on the city's periphery, which is reflected in the city's burden of disease pattern, which follows the quadruple burden of disease pattern observed nationally (10), with high infectious disease mortality among young children; high mortality from violence and injuries among young adults; high non-communicable disease mortality in older age groups; and rising HIV/AIDS mortality among young adults and young children (11). This disease profile is strongly associated with inequality, including for interpersonal violence and injury. For example, the highest homicide rates were recorded in the poorest sub-district of Khayelitsha (120 per 100,000 people), approximately three times the rate recorded in the city centre (42 per 100,000 people) (11).

In recent years, there has been a push to develop locally appropriate injury surveillance efforts in settings that lack conventional surveillance systems due to resource constraints. For instance, hospital-based surveillance projects have been undertaken recently in several LMIC such as Nigeria (12), China (13), and Rwanda (14) according to local needs and capabilities. Epidemiological analyses of injury data collected for these projects have provided evidence of injury patterns, which could be useful for developing evidence-based prevention programmes in the local setting. In addition to its value for epidemiological and prevention purposes, injury surveillance data are also valuable for patient outcome assessment, resource allocation, quality improvement, trauma care systems development, and hospital administration (15), which further demonstrates the huge benefit that injury surveillance could have when implemented in LMIC (16, 17).

This paper presents the epidemiological findings from a 16-month injury surveillance study conducted at Groote Schuur Hospital (GSH) in Cape Town, the main urban centre of Western Cape Province in South Africa. A middle-income country, South Africa is subject to massive rates of injury, which is largely attributed to road-traffic crashes and ‘intentional’ injuries stemming from interpersonal violence (18, 19). Every year, more than 1.5 million people die from violent injuries worldwide while many more are permanently disabled; however, 90% of all cases of interpersonal violence occur in LMIC (20), though rates do vary country by country. South Africa is one of the only countries in the world that has a higher rate of intentional injury than unintentional (e.g. from falls, motor-vehicle collisions) (19). There is a growing body of research on the causes of injury in South Africa that acknowledges the role of alcohol, social anomie, and structural economic effects (21–23). However, the data required to assess temporal and other specific patterns of injury have been absent.

While trauma care is generally very good in South Africa and at GSH in particular, the lack of surveillance data for spatial and temporal analysis of injuries has restricted the ability to design evidence-based interventions – that are place-specific. Data analysis for this study provides clear evidence of injury patterns in this setting, and in particular, the huge burden of intentional injury and violence. Findings of this study could be useful for designing injury prevention efforts that target the root causes of interpersonal violence, which is a priority focus of the Western Cape government's Burden of Disease reduction project (24). Furthermore, the design of the surveillance activities reported on in this paper may provide a blueprint for other health facilities with similar levels of resources to engage in injury surveillance activities.

## Methods

This paper reports the epidemiological findings of a 16-month injury data collection study, which is phase 2 of a larger ongoing surveillance project carried out in the trauma centre of GSH, documented in (16, 17, 25, 26). Phase 1 of the project (26) was an exploratory 1-month data collection pilot study conducted at GSH in October 2008, designed to assess the feasibility of systematic prospective surveillance in this hospital. Prior to the phase 1 pilot study, the trauma centre had attempted to implement a comprehensive trauma registry with a complex data collection form and numerous data fields to collect; however, it was deemed to be overly complicated for this busy hospital setting. In concert with the trauma centre managers prior to phase 1, a needs assessment was conducted which focused on four important considerations for the project, derived from Schultz et al. (27): 1) defining the goals of the surveillance project, 2) defining the inclusion criteria, 3) defining the data fields to be collected, and 4) determining which data collection model to use, either coordinator- or provider-based (PTR). The coordinator-based (CTR) trauma registry model employs a person or persons to complete data forms and manage the registry, while the PTR trauma registry model relies on existing staff and clinicians to complete surveillance forms while attending patients (27). In phase 1: 1) the goal of the project was to explore the feasibility of surveillance at GSH, 2) all admitted patients were included, 3) a small number of fields were collected (a minimal data set), and 4) the CTR data collection model was used (26, 28). In phase 2 of the surveillance project, the four considerations were modified based on the findings of phase 1: 1) since feasibility was ascertained in phase 1, the goal of phase 2 was to engage in longer term surveillance in order to understand the burden of injury in this setting, 2) similar to phase 1, all patients were included, 3) a larger number of fields were collected in phase 2, and 4) the PTR data collection model was used. The use of the PTR model in phase 2 enabled much longer term surveillance, covering 16 months of admissions to the trauma centre, from June 2010 to October 2011.

In phase 2, following the PTR model, clinicians and staff working in the trauma centre collected patient data using a standardized trauma admissions form. The final version of the form was developed based on input from clinical staff and trauma managers, and from the findings of phase 1. To limit the added workload on already busy clinical staff, the data collection form was integrated into the documentation workflow by combining it with the existing patient physical assessment forms. All staff involved in data collection were provided training at the launch of the project, and ongoing education materials were provided throughout phase 2.

These data are thus the accumulation of a series of pilot projects designed and implemented to test the viability of an ongoing surveillance system in a LMIC. Our first pilot project was in 2008. In this paper, we analyse the data from the still paper-based follow-up pilot, from 2010 to 2011 with 16 months of data. Subsequently, we are working on electronic-based trauma surveillance system in the same hospital. It is important to emphasize that the major constraint to an ongoing surveillance system is human resources. The clinicians, registrars, and staff are all stretched thinly. Each of these series of pilots that we implemented at GSH has had the prerequisite of being largely self-funded (through grants and personal research funds).

Trauma data were extracted and analysed using SPSS (Statistics Package for the Social Sciences, version 20). Prior to analysis, records were manually checked for data coding errors, missing records, and logical consistency; 170 cases were removed, resulting in a total of 8,275 valid cases. Variables considered in this analysis are patient age and sex, whether the patient was visibly under the influence of drugs, alcohol, or both at the time of admission, the day and time of incident, the mechanism of injury, patient's spoken language(s), and intent (whether the injury was intentional, unintentional, or of unknown intent). Pearson's chi-squared test was used to identify significant bivariate associations between use/intent, day of week/intent, day of week/alcohol use, and patient language/intent. Student's *t*-test for comparing means was selected to assess the significance of difference for patient mean age between weekdays and weekends. Age- and sex-adjusted logistic regression models were fitted to calculate odds ratios and 95% confidence intervals for the outcome violent or non-violent trauma. Using the maximum number of valid cases for each model, four bivariate-adjusted models for violent trauma (versus non-violent trauma) as the outcome variable were calculated. Selected model predictors were patient sex (5,684 valid cases), alcohol consumption (3,828 valid cases), Xhosa language (5,684 valid cases), and English language (5,684 valid cases). A fifth, multivariate binary logistic regression model was fitted with the following predictors: victim alcohol consumption (binary), Xhosa-speaking (binary), and whether the trauma occurred on the weekend (Friday, Saturday, or Sunday, versus Monday to Thursday), with a total of 3,828 valid cases. The model was also adjusted for patient age and sex, and all predictor and adjustment variables were evaluated for significance using the Wald test, and 95% confidence intervals for the odds ratios were computed.

## Results

As shown in Table 1, of cases with known intent, the majority of cases were recorded as intentional (2,895, or 51%), suggesting a disproportionately high burden of violent trauma; this is likely lower than the actual figure due to underreporting of intimate partner, familial, and gang violence. Of the violent cases where the motive was recorded, 5.5% of injuries were self-inflicted. Nearly three in every four patients is male, with consistently higher proportions of substance use, weekend injury, and weapons-related, intentional trauma, excluding self-harm.

**Table 1:** Number of trauma cases at Groote Schuur Hospital, Cape Town, from October 2010 to September 2011

	Female	Male	Total
	2,305	6,123	8,445
Patient under influence of substance			
Alcohol	326	1,366	1,692
Drugs	4	36	40
None	1,237	2,499	3,736
Day of injury			
Monday to Thursday	1,042	2,365	3,407
Friday to Sunday	1,263	3,758	5,021
Mechanism of injury			
Sharp object	294	1,624	1,918
Blunt object	324	1,205	1,529
Firearm	38	340	378
Bodily force	134	361	495
Motor-vehicle collision	439	916	1,355
Fall	757	828	1,585
Other	139	320	459
Intent			
Intentional	520	2,409	2,936
Unintentional	1,021	1,811	2,838

Note the high proportions of cases occurring on weekends, featuring patient alcohol consumption, and reported as intentional. This suggests a disproportionate burden of violent injury in the study area.

**Fig. 1:**
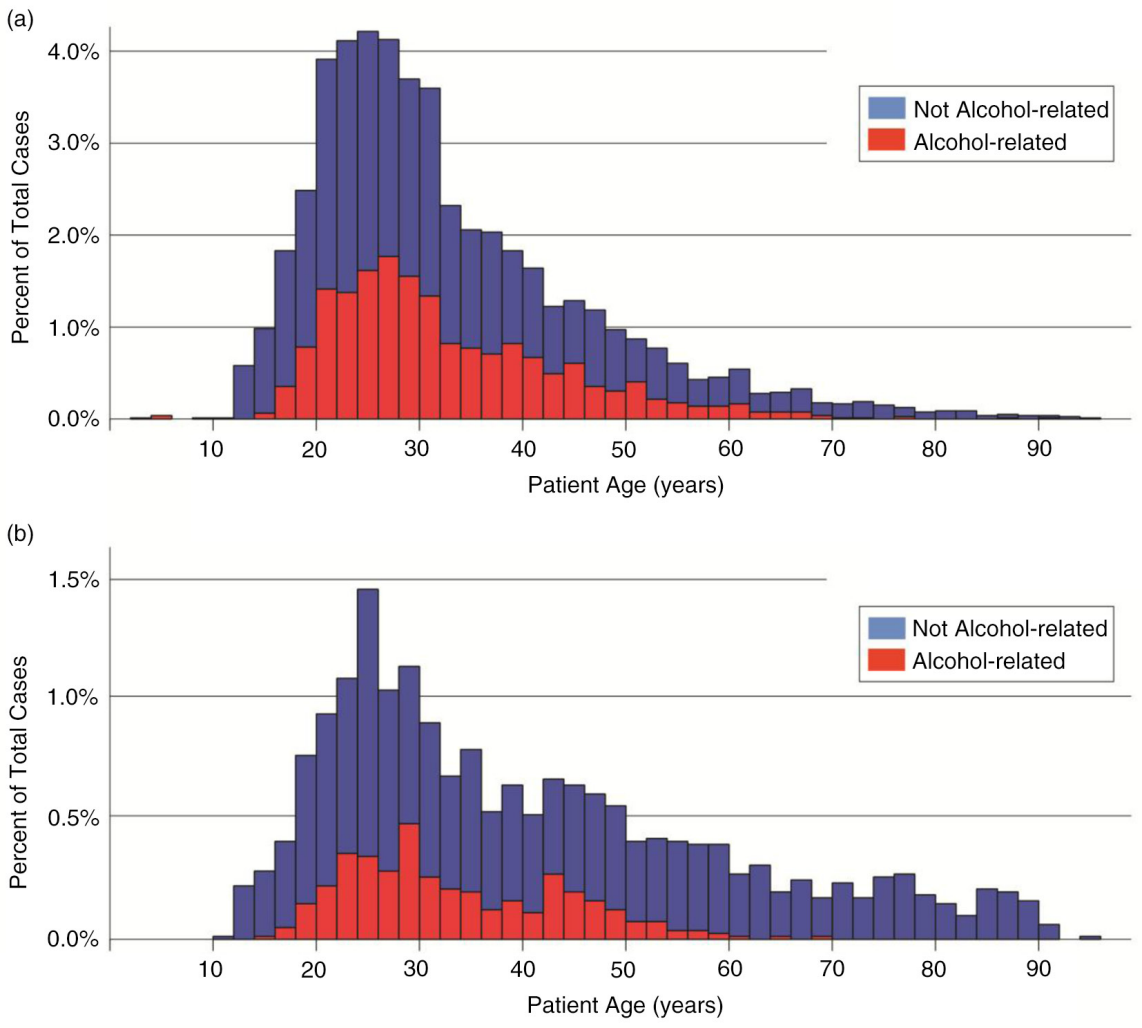
Age distributions of trauma admissions, by sex. A smaller ratio of male-to-female patients is observed among cases where the patient was not under the influence of alcohol. In alcohol-related cases, (a) male patients are consistently more prevalent than (b) female patients, with a less prominent peak in distribution.

When contrasted by patient sex (Fig. 1), the age distribution highlights a high ratio of male-to-female cases from ages 18 to 35, diminishing to become equal among patients ages 65+. Male odds (age-adjusted) of having been injured in a violent incident are 2.2 times those of a female in these data (95% CI: 1.95, 2.51), a highly significant difference.

**Fig. 2:**
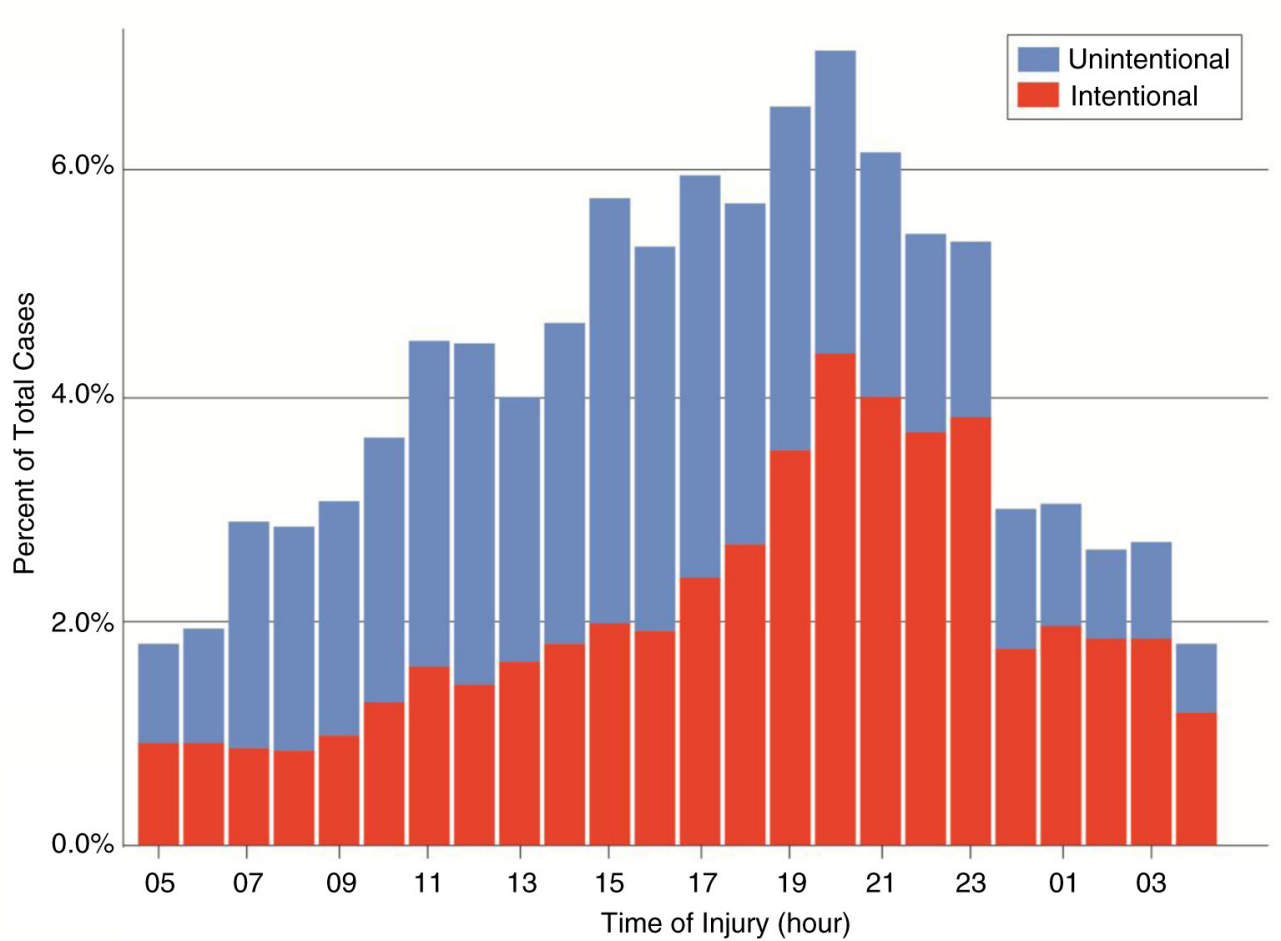
Number of trauma cases by time of day and intent. While the number of violent trauma cases increases through the afternoon and evening, the number of unintentional injuries remains consistent through daylight hours. Note that cases where the intent was unknown are not represented in this figure.

A temporal component to trauma admissions is evident in Fig. 2, where a distinctive increase in violent injury is observed in the evening. This pattern appears to differ by intent, such that a greater proportion of intentional injuries occur during the night, while unintentional injury peaks late in the afternoon hours.

**Fig. 3:**
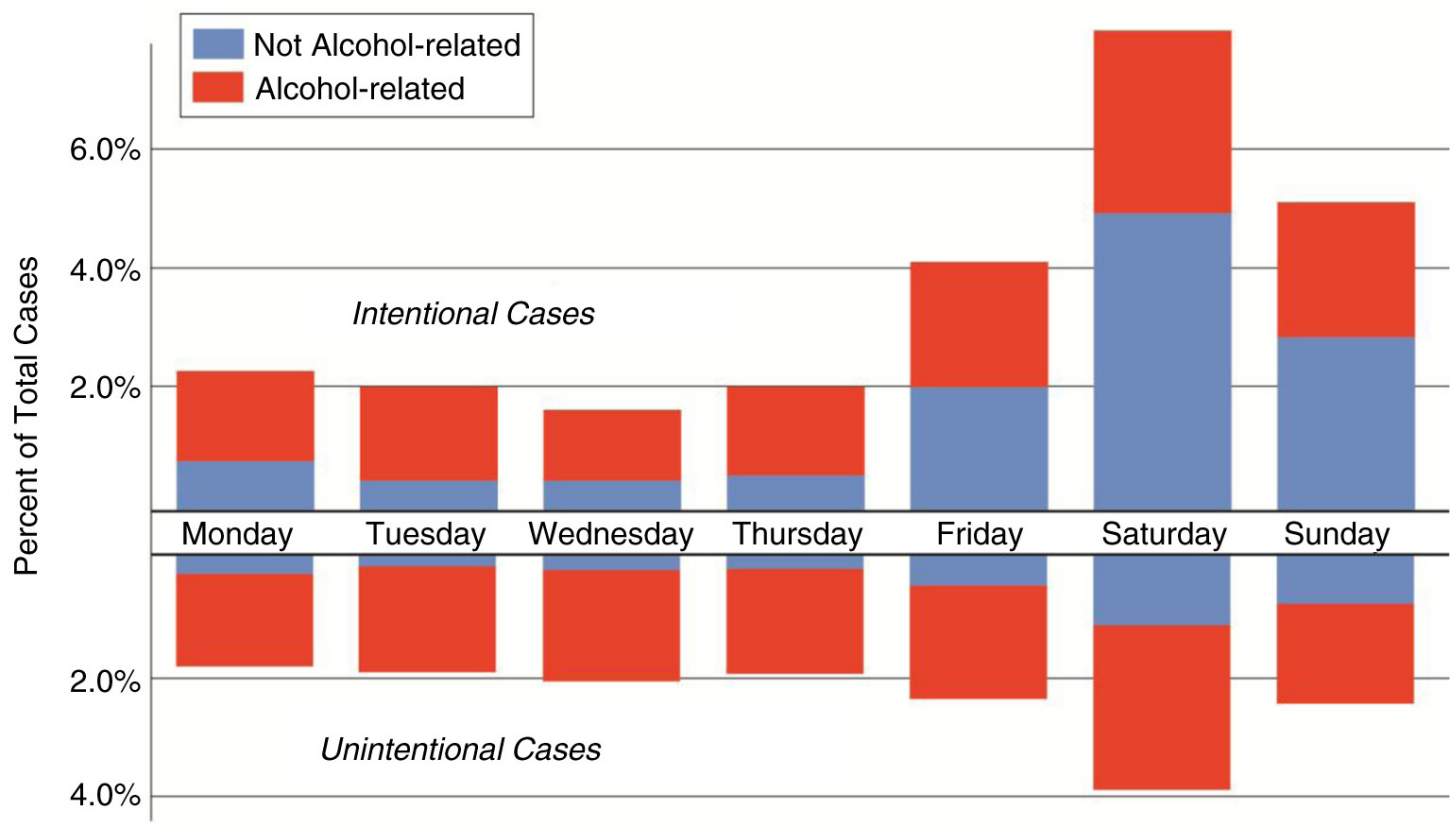
While the number of intentional injuries among trauma admissions not under the influence of alcohol is relatively consistent throughout the week, this effect among alcohol-related cases is pronounced. Unexpectedly, a greater proportion of patients under the influence of alcohol were unintentionally injured during the weekend.

Two-thirds of all violent trauma cases were found to have occurred on the weekend (including Friday), as illustrated in Fig. 3. This is a slightly higher proportion than unintentional injuries, approximately 60% of which occur on these 3 days.

The influence of substance use is evidenced in Fig. 3, where the number of patients visibly under the influence of alcohol at the time of admission increases significantly on Friday, Saturday, and Sunday (*x*^2^=296.14, *p*<0.0005). Notably, the mean patient age significantly decreases during these days, compared to the weekday average (mean difference=2.45 years, *t*=7.002, *p*<0.0005).

**Fig. 4:**
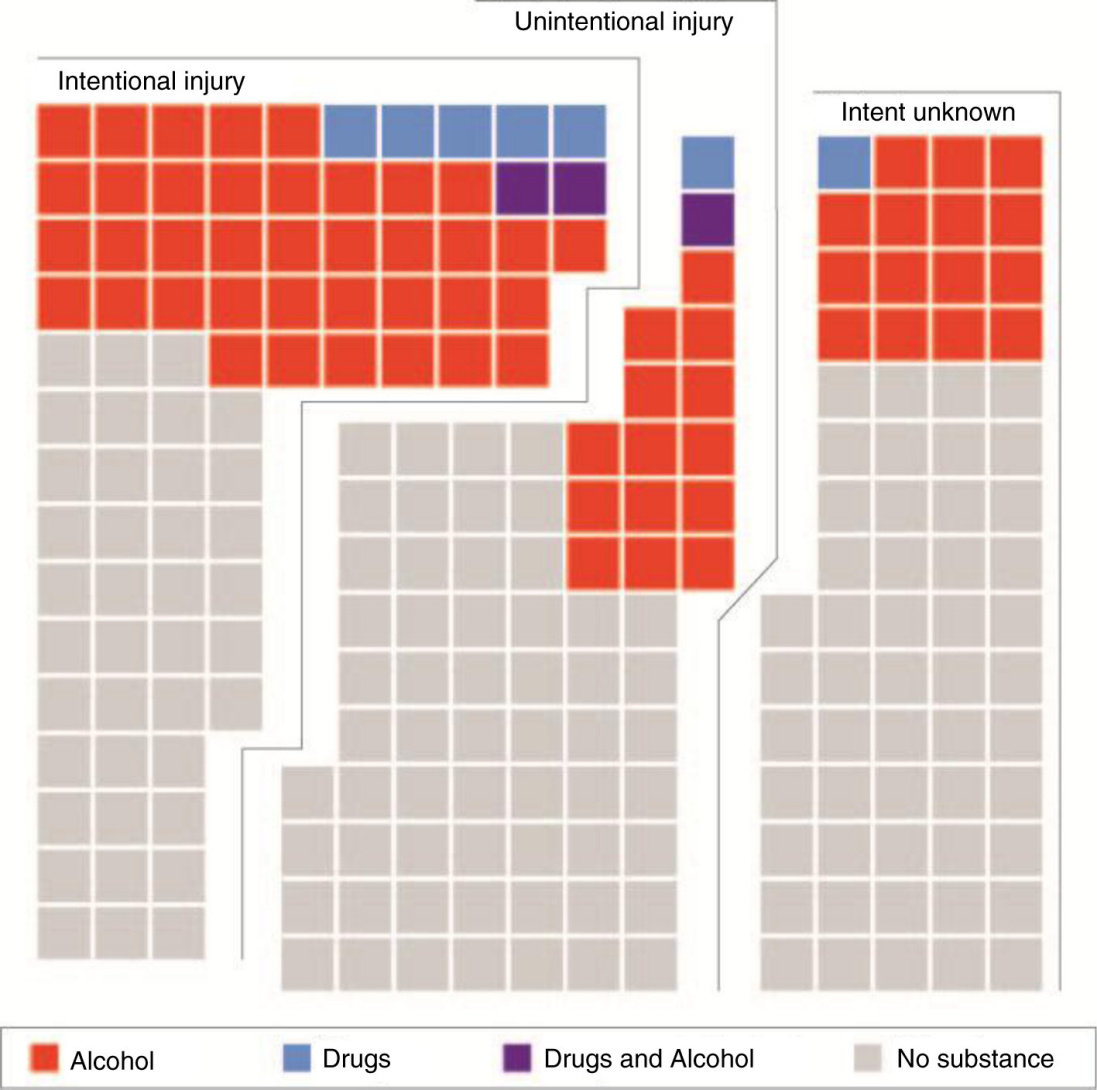
Comparison of cases by patient substance use and intent. Each square represents approximately 40 patients. The higher proportion of patient drug and/or alcohol use among intentional injuries underscores the significant role of alcohol in interpersonal violence.

As shown in Fig. 4, patient drug and/or alcohol consumption are significantly associated with intent. Among cases where the patient was recorded as being under the influence of alcohol, 72% were reported as intentional injury. Within these data, the odds of a patient under the influence of alcohol having been injured in a violent incident are 3.47 times higher (age- and sex-adjusted) than one who was not recorded as under the influence (95% CI: 3.00, 4.02). However, the true proportion is likely higher due to underreporting and may be masked within cases where the intent is recorded as unknown. Typically, drug use is even more underreported and therefore underrepresented.

A strong disparity between patient sexes was also observed, as 81% of patients under the influence of alcohol (*x*^2^=101.53, *p*<0.0005) and 90% of patients under the influence of drugs (*x*^2^=29.12, *p*<0.0005) were male.

Patient language was also found to be significantly associated with violent trauma. Within these data, Xhosa-speaking patients’ odds of having being injured in a violent incident were 2.46 times (age- and sex-adjusted) those of non-Xhosa speakers (95% CI: 2.16, 2.80), a highly significant difference. Conversely, English speakers were primarily injured in non-violent incidents, with a significantly low odds ratio of 0.41 (95% CI: 0.37, 0.46, adjusted for age and sex).

The multivariate model demonstrated significantly elevated odds of violent trauma for all predictor variables, shown in Table 2. That these patterns persist when observed in combination with all other predictors underscores the strength of alcohol consumption, patient sex, weekend/weekday trauma, and victim language as covariates of violent trauma in our study area. Significance of these variables in both the bivariate models and the multivariate model suggests independent effects of the selected predictors as risk factors. Alcohol consumption remains the strongest categorical predictor variable, even when controlling for all other selected variables. The effect of age and sex are also persistent, with a higher risk among males, and among younger patients. Overall model fit was strong, with 65% overall predictive accuracy and high significance (*p*<0.001).

**Table 2:** Multivariate binary logistic regression model for violent trauma, using only cases with complete information for model variables (*N*=3,828)

	Beta coefficient	Std. error	*p* (Wald)	Odds ratio	95% CI (OR)
Intercept	−0.394	0.127	0.002	0.068	
Alcohol consumed	1.102	0.078	<0.0005	3.009	2.581, 3.508
Xhosa-speaking	0.78	0.085	<0.0005	2.181	1.847, 2.575
Weekend (Fri/Sat/Sun)	0.256	0.074	0.001	1.292	1.117, 1.493
Age (years)	−0.021	0.003	<0.0005	0.979	0.974, 0.984
Sex (male)	0.674	0.081	<0.0005	1.963	1.674, 2.302

## Discussion

Given the scarcity of comprehensive ongoing injury surveillance in this and many other settings in LMIC, the findings of this study present a rare glimpse into the specific, data-based, patterns of non-fatal injury injuries in Cape Town. While researchers have illustrated the high burden of intentional injury in South Africa based on estimates and mortality records (e.g. 18, 28), longer term prospective epidemiological analyses of both injury morbidity and mortality provide a more valuable and comprehensive picture of injury burden. Analysis of phase 2 data set has illuminated some interesting patterns of injury that could be useful for designing local injury prevention initiatives. Most notably, these data provide valuable insight into the interrelated connections between intentional injury and violence, age, sex, and substance abuse in Cape Town. While many of these connections have been suspected, a corpus of data demonstrating them has been absent.

Significantly, just over 50% of GSH patients present to the trauma centre due to an act of violence or self-harm (assuming that the intentional/unintentional rate holds for the unrecorded fields). Looking more closely reveals important considerations for violence prevention. Overall, the data illustrate a much higher rate of injuries in males, especially in younger age groups. This finding is even more acute in terms of violence-related injury specifically, in which males are almost three times more likely to be the victim. Clearly, violence prevention initiatives that target this demographic may be beneficial in reducing this burden; however, it must also be noted that these figures could possibly underrepresent the number of female victims of domestic violence given cultural beliefs, potential for stigmatization, and the normalization of violence that restrict the reporting this type of violence in this and many other societies (29). The significant increase in substance use especially among younger patients on the weekend (Friday to Sunday) is notable, especially given that a majority of intentional injuries occurred at this period, and a vast majority of patients under the influence of drugs or alcohol were male. These interconnected findings provide substantial evidence of an important etiological agent of violence-related injury in this context, and a specific target for intervention: younger males under the influence of alcohol and drugs especially at weekends. These findings provide longer term evidence to back up a previous short-term cross-sectional patient survey conducted at a Cape Town hospital, which found that in the year 2000, 65% of patients being treated for violence-related injuries tested positive for alcohol use, and that greater than two-thirds of patients were males in their early 30s (30).

Violence is increasingly recognized as a priority area for public health policy, especially since the release of the 2002 *World Report on Violence and Health* (31), which identified a need to characterize violence as a public health issue embedded in social, political, and economic contexts (32). Ongoing hospital-based injury surveillance systems represent one of the most effective means of characterizing these specific contexts, allowing for evidence-based prevention programmes to be designed. A review by Matzopoulos et al. (24) identified a range of interventions that could be enacted to reduce the burden of violence in Western Cape Province, which target either *biological*, *behavioural*, *societal*, or *structural* risk factors. In the case of the Cape Town study area specifically, interventions that target the younger male demographic and access to alcohol and other substances might be effective strategies. Although all four categories play a role in violence-risk, intervening at the more midstream and upstream levels (community, societal, structural, and policy) might have greater sustained success, despite being more difficult to measure compared with interventions aimed at addressing downstream individual-level behaviours. For instance, some effective and promising upstream interventions for violence prevention in the Western Cape identified by Matzopoulos et al. (24) include reducing income inequality and other social determinants of health, improving the criminal justice and social welfare systems, restricting access to firearms and alcohol, developing education and awareness campaigns, and changing cultural norms that support aggression and violent behaviours.

Although quantitative evidence derived from prospective surveillance is the benchmark in injury prevention, combining it with other sources and types of data is particularly valuable for unearthing the root causes for the patterns observed and potential solutions. For those observed in this study, a suitable next step would be to conduct qualitative surveillance including interviews and focus groups with the targeted demographic and those affected by violence. Makanga et al. (under review) carried out focus groups in five Cape Town communities with high rates of violence to identify perceptions of local environmental risk factors for intentional injury. Although this study was small-scale and cross-sectional, the findings resonate with the present study, and together they could provide both robust quantitative evidence of injury patterns and more locally specific evidence that could inform mid- and upstream interventions. Future research in this area should explore the benefits of combining quantitative injury surveillance with place- or population-specific qualitative research in order to tease out the individual, community, and policy-related factors that could be modified to reduce the toll of violence and intentional injury.

### Limitations

This study has several limitations. The first is based on the quality and consistency of data collection. The data were collected by Doctors and Registrars as patients are admitted and during rounds. There is little or no institutional support for these data collections; as a result, the data are sometimes incomplete. In some cases, those entering the data make executive decisions about the relative importance of a field. These decisions will be varied depending on the context, and the specific case. We cleaned the data extensively in the interests of maintaining integrity. However, if we had removed all cases with one or more fields missing, we would have eliminated fully half of the records. In the vast majority of cases, only minor fields (often relating to specific diagnosis) were missing. Given that we assessed many records on an individual basis – to determine reliability – and eliminated over 2,000 records, we believe that the results are reliable in the context of the broader findings (e.g. sex and temporal patterns as well the high rates of intentional injury).

Although it is expected that the surveillance activities accounted for the vast majority of patients accessing the trauma centre at GSH, not all trauma patients in Cape Town use this hospital. It is possible that the patterns observed in this data set are specific to the patient population of the GSH catchment. As such, data from the other major trauma centre in Cape Town, Tygerberg hospital should also be analysed to provide more complete evidence for decision makers to design prevention activities for all of Cape Town. Future research should seek to develop an integrated city-wide surveillance system. Moreover, in Cape Town as in other settings, a proportion of injured persons do not access trauma care at all. Although the findings of the study highlight violence as a component of injury in this setting, it is likely that familial and intimate-partner violence has been underreported. Novel approaches should be designed to uncover the true burden of this type of violence, since hospital-based methods are frequently unable to fully account for it.

## Conclusion

The findings of this 16-month surveillance project provide valuable evidence of the patterns of injury in Cape Town, and in particular the high rate of intentional injuries. This confirms anecdotal reports and indications from smaller scale surveillance activities and estimates. Analysis of the data set has identified specific targets for intentional injury and violence prevention. Most notably, interventions should be designed that target the intersection of violence among young males, the weekend time period, and substance abuse. Furthermore, the evidence provided here demonstrates the value in developing systematic injury surveillance activities that are locally appropriate and feasible, to understand the local injury context and to develop evidence-based interventions. Future data collection undertakings that include mortality figures are strongly urged in the effort to base injury prevention on surveillance in low- and middle-income countries.
